# Model Progress for Tensile Power of Polymer Nanocomposites Reinforced with Carbon Nanotubes by Percolating Interphase Zone and Network Aspects

**DOI:** 10.3390/polym12051047

**Published:** 2020-05-02

**Authors:** Yasser Zare, Kyong Yop Rhee

**Affiliations:** Department of Mechanical Engineering, College of Engineering, Kyung Hee University, Yongin 446-701, Korea; y.zare@aut.ac.ir

**Keywords:** polymer CNT nanocomposites, mechanical percolation, interphase properties, filler network, tensile strength

## Abstract

In the present work, a simple simulation is advanced based on a Callister equation considering the impacts of interphase and carbon nanotube (CNT) nets on the strength of nanocomposites after percolation onset. The advanced model can analyze the strength of nanocomposite by filler aspect ratio (*α*), percolation beginning ($\varphi_{p}$), interphase depth (*t*), interphase power (*σ_i_*), net density (*N*), and net power (*σ_N_*). The empirical consequences of several samples agree with the estimations of the industrialised model. The nanocomposite strength straightly depends on “*α*”, “*t*”, “*σ_i_*”, “*N*”, and “*σ_N_*”, while the radius and percolation onset of CNT play the inverse characters. The reasonable impacts of net and interphase possessions on the nanocomposite strength rationalise the accurate progress of the Callister equation.

## 1. Introduction

Many researches have been conducted on carbon nanotubes (CNT) and polymer CNT nanocomposites, because of their large aspect ratio, low density, acceptable chemical stability, excellent thermal conductivity, and mechanical possessions [1,2,3,4,5,6,7,8,9,10,11,12,13,14]. Therefore, CNT show a good potential in polymer nanocomposites, because the various defects of neat polymers limit their applications in different fields. However, some challenges occur in the synthesis of polymer CNT nanocomposites. For example, van der Waals attractions create aggregates/agglomerates during the fabrication of nanocomposites [15,16,17]. The CNT can be modified with strong acid or strong oxidization agents to disrupt the agglomerates and provide the noble dispersal of filler at the nanoscale in the polymer medium, which are obligatory to develop the sample properties [18,19,20]. 

The big nanoparticles surface part per volume and the robust linkage at the matrix–nanoparticles interfacial interface construct the interphase zone in the nanocomposites [21,22,23,24]. The properties of interphase such as depth, power, and modulus were investigated in the previous papers [25,26]. It was revealed that a dense and sturdy interphase causes a substantial modulus and power in polymer nanocomposites [27]. The simulation papers for the mechanical possessions of nanocomposites simplify the characterization of interphase, because the experimental handling of interphase involves the characterization of interfacial interaction at the nanoscale [28,29,30].

Polymer CNT nanocomposites generally display high conductivity whenever the CNT volume portion is higher than the percolation beginning [31,32]. In fact, the percolation onset is the smallest portion of nanofiller in the nanocomposites, which can form the net structures. So, it is impossible to gain the electrical conductivity in an insulated polymer below the percolation threshold, because the network of nanoparticles can conduct the electric current. It can be concluded that the electrical percolation onset is the critical content of nanofiller improving the electrical conductivity, meaningfully.

By the addition of filler concentration in polymer nanocomposites, a significant increment was described for mechanical possessions referred to as mechanical percolation onset [33,34]. The quick growths of mechanical powers is scarcely explained by electrical percolation beginning, but both electrical and mechanical percolations were correlated to the size of nanoparticles [35,36]. The mechanical percolation was also explained in the literature. Ouali et al. [37] established an equation for the stiffness of polymer composites supposing the filler percolation. It was widely utilized by the previous researchers to estimate the nanocomposites modulus after percolation inception [38,39]. However, Ouali and other conventional models cannot estimate the mechanical performance of nanocomposites, because they do not reflect the excellent possessions of nanoparticles such as big surface area and networking capability. It was reported that the nanoparticles such as CNT produce the net in the nanocomposite samples at very small filler loadings [40,41,42]. This means that the percolation threshold for conductivity and mechanical behavior commonly happens in polymer CNT nanocomposites.

The simple Callister model [43] disregards the interphase and net features for the tensile/yield strength of nanocomposites. In this paper, the Callister model is expanded by interphase and filler network characteristics for CNT-reinforced samples. Two interphase and network terms are defined to assume the interphase and network properties. The experimental results of various samples are used to assess the calculations of the original and industrialized equations. Also, the established equations are applied to display the dependencies of interphase and network terms as well as the nanocomposite strength on the nanoparticle size, percolation onset, interphase depth, and network properties.

## 2. Theoretical Views

The tensile strength of nanocomposites is a function of material possessions and interface interaction according to the Callister model [43] as:

(1) $$\sigma_{R}=1+(\frac{\alpha s}{\sigma_{m}}-1)\varphi_{f}{}^{}$$

where “*σ_R_*” shows the relative strength (*σ_R_* = *σ_c_*_/_*σ_m_*), “*σ_c_*” and “*σ_m_*” denote the strengths of nanocomposite and polymer medium, individually. “*α*” is filler aspect ratio defined as *α* = *l*/*d*; “*l*” and “*d*” are the filler length and diameter, in that order. Also, “*S*” is an interfacial stress transfer factor demonstrating the extent of interfacial linkages and “$\varphi_{f}$” is filler volume portion.

Pukanszky [44] also simulated the strength of polymer nanocomposites as:

(2) $$\sigma_{R}=\frac{1-\varphi_{f}}{1+2.5\varphi_{f}}\exp(B\varphi_{f})$$

where “*B*” as interfacial parameter reflects the capacity of stress transferring from matrix toward nanoparticles. “*B*” is a function of the depth and power of interphase district as:

(3) $$B=(1+A_{c}d_{f}t)\ln(\frac{\sigma_{i}}{\sigma_{m}})$$

where “*A_c_*” and “*d_f_*” are filler specific surface area and density, correspondingly. Also, “*t*” and “*σ_i_*” show the depth and strength of interphase region. So, “*B*” parameter can express the possessions of interphase in the nanocomposites by the strength experimental measurements.

Pukanszky model can be restructured to:

(4) $$\ln(\sigma_{R}\frac{1+2.5\varphi_{f}}{1-\varphi_{f}})=B\varphi_{f}$$

where the plot of $\ln(\sigma_{R}\frac{1+2.5\varphi_{f}}{1-\varphi_{f}})$against “$\varphi_{f}$” results in a linear correlation with slope of “*B*”.

In our previous work [45], it was shown that the “*B*” parameter for polymer CNT nanocomposites can be expressed by:

(5) $$B=(1+\frac{2t}{R})\ln(\frac{\sigma_{i}}{\sigma_{m}})$$

where “*R*” is the radius of nanotubes. Similarly, it was reported that the maximum level of “*B*” as “*B*_max_” can be given by:

(6) $$B_{\max}=(1+\frac{80}{R})\ln(\frac{25000}{\sigma_{m}})$$

where “*R*” and “*σ_m_*” get nm and MPa units, correspondingly.

“*B*” parameter was also related to “*S*” for the nanocomposites containing long fillers such as clay and CNT [46] as:

(7) $$B=\frac{\alpha s}{\sigma_{m}}+2.4$$

By restructuring of above equation, the “*S*” parameter is calculated by:

(8) $$s=\frac{(B-2.4)\sigma_{m}}{\alpha }$$

The “*S*” parameter can be expressed by the interphase properties through the replacement of “*B*” from Equation (7) in the above equation as:

(9) $$s_{I}=\frac{(1+\frac{2t}{R})\ln(\frac{\sigma_{i}}{\sigma_{m}})\sigma_{m}-2.4\sigma_{m}}{\alpha }$$

Also, the “*S*_max_” parameter can be given assuming the “*B*_max_” as:

(10) $$s_{\max}=\frac{(1+\frac{80}{R})\ln(\frac{25000}{\sigma_{m}})\sigma_{m}-2.4\sigma_{m}}{\alpha }$$

In our previous study [45], the “*B_N_*” parameter was defined to capture the network role in Pukanszky model as:

(11) $$B_{N}=\frac{\alpha N}{10^{5}}\ln(\frac{\sigma_{N}}{\sigma_{m}})$$

where “*N*” is the CNT number in a unit part as network density and “*σ_N_*” is the network power.

The “*S*” parameter can be also expressed by the properties of filler network. In this regard, the “*B_N_*” definition is replaced from Equation (11) into Equation (8) as:

(12) $$s_{N}=\sigma_{m}\frac{N}{10^{5}}\ln(\frac{\sigma_{N}}{\sigma_{m}})-\frac{2.4\sigma_{m}}{\alpha }$$

When the interphase and net effects are taken into account in the Callister model (Equation (1)), a developed model is obtained as:

(13) $$\sigma_{R}=1+(\frac{\alpha s_{I}}{\sigma_{m}}+\frac{\alpha s_{N}}{\sigma_{m}}-1)\varphi_{f}{}^{}$$

By substituting of “*S_I_*” and “*S_N_*” parameters from Equations (9) and (12) into above equation, the developed Callister model is proposed as:

(14) $$\sigma_{R}=1+[(1+\frac{2t}{R})\ln(\frac{\sigma_{i}}{\sigma_{m}})+\alpha \frac{N}{10^{5}}\ln(\frac{\sigma_{N}}{\sigma_{m}})-5.8]\varphi_{f}{}^{}$$

which expresses the strength of nanocomposites by interphase and net characteristics.

As indicated, the percolation for electrical conductivity and rigidity of nanocomposites are adversely correlated to “*α*”. Chen et al. [47] suggested the percolation onset for the stiffness of CNT net as a function of “*α*” as:

(15) $$\varphi_{p}=\frac{2.2}{\alpha }$$

By this expression, the “*S_N_*” parameter (Equation (12)) can be given by percolation threshold as:

(16) $$s_{N}=\sigma_{m}\frac{N}{10^{5}}\ln(\frac{\sigma_{N}}{\sigma_{m}})-\frac{1.2\sigma_{m}\varphi_{p}}{1.1}$$

Similarly, the “$\varphi_{p}$” significance on the nanocomposites strength can be evaluated by replacing of “*S_N_*” from the latter equation into Equation (13) as:

(17) $$\sigma_{R}=1+[(1+\frac{2t}{R})\ln(\frac{\sigma_{i}}{\sigma_{m}})+\frac{2.2N}{10^{5}\varphi_{p}}\ln(\frac{\sigma_{N}}{\sigma_{m}})-5.8]\varphi_{f}{}^{}$$

which reveals the correlations of nanocomposites strength to the dimension and percolation onset of CNT in addition to the physical characteristics of filler net and interphase.

## 3. Results and Discussion

Firstly, the predictability of the original and industrialised models are assessed by various experimental results from literature. After that, the effects of interphase and net possessions on the “*S_I_*” and “*S_N_*” and the strength of nanocomposites are designed by 3D and contour plots using MATLAB software.

**Figure 1:**
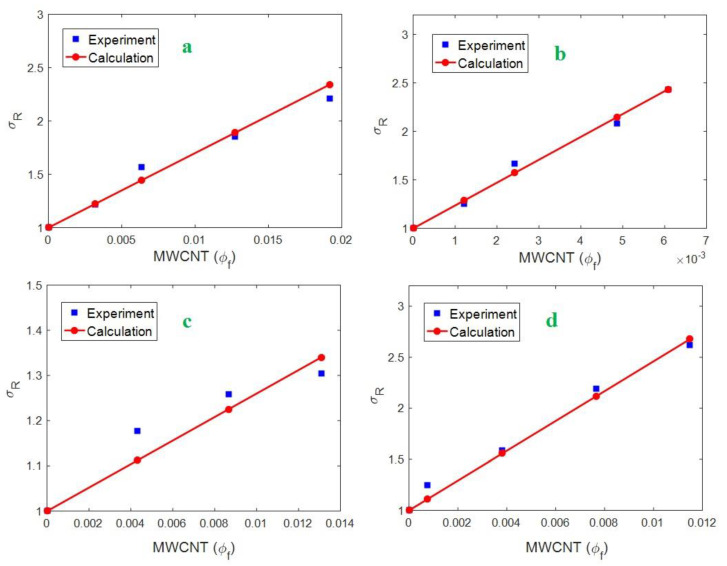
The experimental relative strength and the calculations of Callister model for (**a**) PAN/MWCNT, (**b**) PSQ/MWCNT, (**c**) PP/MWCNT and (**d**) chitosan/MWCNT examples.

Figure 1 exhibits the experimental results of relative strength for polyacrylonitrile (PAN)/multi-walled CNT (MWCNT) nanofiber (*σ_m_* = 70 MPa) [48], polysilsesquioxane (PSQ)/MWCNT (*σ_m_* = 6 MPa) [49], PP/MWCNT (*σ_m_* = 28.2 MPa) [50] and chitosan/MWCNT (*σ_m_* = 11.6 MPa) [51] and the calculations of original Callister model at different filler volume fractions. It is observed that the calculations correctly fit the experimental outputs, which authorize the general rationality of the Callister model. However, the slopes of the lines for PAN/MWCNT, PSQ/MWCNT, PP/MWCNT, and chitosan/MWCNT samples are calculated as 70, 236, 26, and 146, respectively, which are equal to$\left(\frac{\alpha s}{\sigma_{m}}-1\right)$according to Equation (1). Assuming the “*σ_m_*” values of samples and an average α = 300, the “*S*” is calculated as 16.6, 4.74, 2.54, and 5.7 MPa for PAN/MWCNT, PSQ/MWCNT, PP/MWCNT and chitosan/MWCNT samples, respectively. Nevertheless, Equation (10) obtains the “*S*_max_” values for PAN/MWCNT, PSQ/MWCNT, PP/MWCNT and chitosan/MWCNT samples as 11.8, 1.5, 5.5, and 2.6 MPa, respectively (average *R* = 10 nm). The “*S*_max_” is the maximum level of “*S_I_*”, which is calculated by the highest ranges of interphase properties.

While the “*S*” values are higher than the “*S*_max_” calculations, a different strengthening agent beside interphase as filler network is effective in these samples. In other words, the filler net plays a strengthening role in polymer nanocomposites alongside the interphase. Therefore, the developed Callister model (Equation (14)) can express the tensile strength of samples and the original model cannot predict the strength above percolation threshold. The satisfactory predictability of the developed model is due to the suppositions of interphase and filler network by “*S_I_*” and “*S_N_*” parameters, while the original model only accounts the interphase belongings.

**Figure 2:**
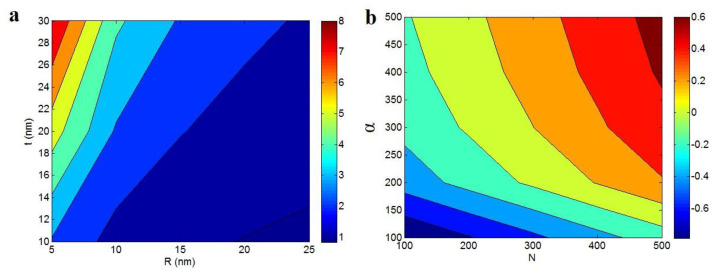
The contour plots for (**a**) “*S_I_*” as a function of “*R*” and “*t*” parameters at *σ_m_* = 40 MPa, α = 300 and *σ_N_* = 5000 MPa and (**b**) dependence of “*S_N_*” on “*N*” and “*α*” parameters at *σ_m_* = 40 MPa and *σ_N_* = 5000 MPa.

Figure 2a illustrates the influences of “*R*” and “*t*” terms on the “*S_I_*” at *σ_m_* = 40 MPa, α = 300 and *σ_N_* = 5000 MPa. The “*S_I_*” values of about 1 are calculated at the high levels of “*R*” and small “*t*”. It mentions that the big particles and thin interphase reduce the “*S_I_*” in nanocomposites. Also, the smallest nanoparticles as well as the densest interphase produce the best “*S_I_*” level as 8. Therefore, the small nanoparticles and dense interphase are wanted for a good level of “*S_I_*” parameter. On the other hand, Figure 2b shows that the “*N*” and “*α*” parameters directly change the level of “*S_N_*” calculated by Equation (12) at *σ_m_* = 40 MPa and *σ_N_* = 5000 MPa. The negative levels of “*S_N_*” are observed by the low ranges of “*N*” and “*α*”, whereas the highest “*S_N_*” is achieved by the great levels of these factors. Hence, the higher levels of “*N*” and “*α*” are required for a higher “*S_N_*” in the nanocomposites.

**Figure 3:**
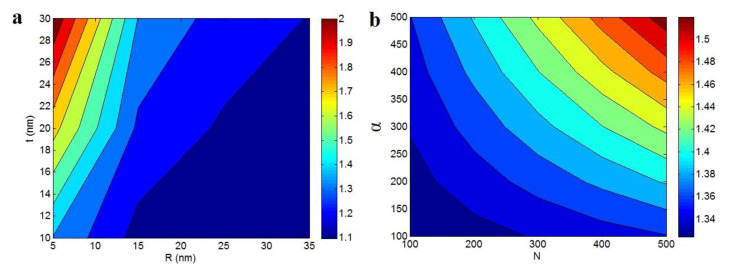
Dependence of “*σ_R_*” on (**a**) nanoparticle and interphase dimensions ($\varphi_{f}$ = 0.02, *σ_m_* = 40 MPa, *α* = 300, *N* = 300 and *σ_i_* = *σ_N_* = 5000 MPa and (**b**) “*N*” and “*α*” parameters at $\varphi_{f}$ = 0.02, *σ_m_* = 40 MPa, *R* = 10 nm, *t* = 20 nm and *σ_i_* = *σ_N_* = 5000 MPa.

Figure 3 also shows the significances of different parameters attributed to interphase and filler net on the relative strength. Figure 3a illustrates the roles of “*R*” and “*t*” parameters as the dimensions of nanoparticles and interphase in the relative strength at $\varphi_{f}$ = 0.02, *σ_m_* = 40 MPa, α = 300, N = 300, and *σ_i_* = *σ_N_* = 5000 MPa. The greatest strength is expectedly observed by the minimum “*R*” and the uppermost “*t*” demonstrating that the thinnest nanotubes and the thickest interphase cause the highest “*σ_R_*” in polymer CNT nanocomposites. Furthermore, the poorest strength is revealed at dense nanoparticles and reedy interphase. The slight diameter of CNT can strengthen the nanocomposites, because the small nanofiller provides the robust and huge interfacial district with the polymer chains, which significantly improve the tensile strength [52,53]. On the other hand, it is clear that a profuse interphase yields a high content of interphase in the samples, which powerfully strengthens the nanocomposites [54,55]. As a result, a better strength owing the thinner nanotubes and the thicker interphase are expected in polymer nanocomposites containing CNT.

Figure 3b also reveals the influences of “*N*” and “*α*” factors on the relative tensile strength at $\varphi_{f}$ = 0.02, *σ_m_* = 40 MPa, *R* = 10 nm, *t* = 20 nm and *σ_i_* = *σ_N_* = 5000 MPa. The “*σ_R_*” level of about 1.32 is detected at the slight heights of both “*N*” and “*α*”, but the maximum relative strength as 1.52 is achieved by N = α = 500. So, the finest level of strength is found by the highest levels of “*N*” and “*α*” parameters. These results are anticipated, because the “*N*” and “*α*” parameters demonstrate the network density and aspect ratio of CNT in nanocomposites. The high ranges of “*N*” demonstrate the great density of CNT network, which strongly strengthens the nanocomposites, due to the excellent tensile strength of CNT as 10–50 GPa. Also, a high “*α*” shows the long and thin nanotubes incorporated in the polymer matrix. Obviously, the longer and thinner nanotubes play a better strengthening role in the nanocomposites, because of the greater and stronger interfacial districts [56]. Therefore, a greater “*α*” rationally causes a higher strength in polymer nanocomposites. The reasons for the impacts of different interphase and network parameters on the polymer nanocomposites strength justify the right expansion of the Callister model.

**Figure 4:**
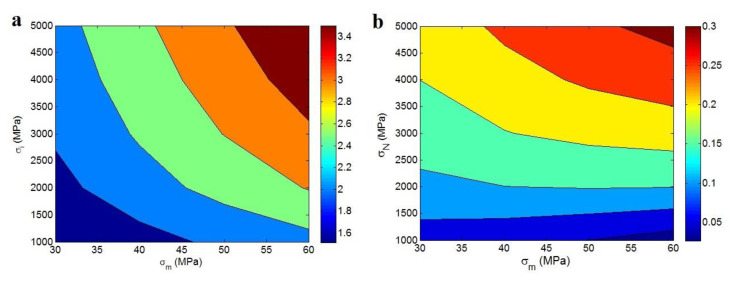
Illustration of (**a**) the influences of “*σ_m_*” and “*σ_i_*” parameters on “*S_I_*” at *R* = 10 nm, *t* = 20 nm and *α* = 300 and (**b**) the roles of “*σ_m_*” and “*σ_N_*” in “*S_N_*” at *N* = 300 and *α* = 300.

Figure 4a represents the “*σ_m_*” and “*σ_i_*” effects on the “*S_I_*” at *R* = 10 nm, *t* = 20 nm, and *α* = 300 according to Equation (9). The high ranges of “*σ_m_*” and “*σ_i_*” increase the “*S_I_*”, where the low levels of these parameters decrease it. The best “*S_I_*” as 3.5 is obtained by *σ_m_* = 60 MPa and *σ_i_* = 5000 MPa, but “*S_I_*” decreases to 1.5 at *σ_m_* = 30 MPa and *σ_i_* = 1000 MPa. Consequently, “*σ_i_*” shows a positive character in “*S_I_*” parameter, because the “*S_I_*” as an interphase parameter directly relates to the strength of interphase. Moreover, Figure 4b displays the dependence of “*S_N_*” on “*σ_m_*” and “*σ_N_*” parameters at N = 300 and α = 300. Generally, “*S_N_*” is directly correlated to both “*σ_m_*” and “*σ_N_*” parameters in polymer nanocomposites. In other words, the “*S_N_*” as a network parameter directly depends on the strengths of polymer medium and nanoparticles net. The greatest “*σ_m_*” and “*σ_N_*” ranges introduce the highest level of “*S_N_*”, while a low “*S_N_*” is observed at low “*σ_N_*”. So, a low level of network strength results in a poor “*S_N_*” at dissimilar values of matrix strength. This trend is sensible, because a poor network should give a small level of network parameter based on the definition of “*S_N_*”.

**Figure 5:**
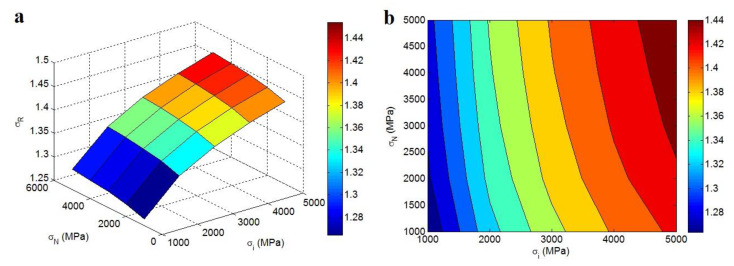
(**a**) 3D and (**b**) contour plots for the “*σ_i_*” and “*σ_N_*” effects on the “*σ_R_*” at $\varphi_{f}$ = 0.02, *σ_m_* = 40 MPa, *R* = 10 nm, *t* = 20 nm, *α* = 300 and *N* = 300.

Figure 5 displays the influences of “*σ_i_*” and “*σ_N_*” factors on the relative strength at $\varphi_{f}$ = 0.02, *σ_m_* = 40 MPa, *R* = 10 nm, *t* = 20 nm, *α* = 300, and *N* = 300 by 3D and contour illustrations. The highest strength is obtained by the highest levels of interphase and network strengths. In addition, the lowliest strength is observed by the smallest values of “*σ_i_*” and “*σ_N_*” parameters. At the same conditions, the relative strength improves from 1.26 at *σ_i_* = *σ_N_* = 1000 MPa to 1.44 at *σ_i_* = *σ_N_* = 5000 MPa, which reveals that the sample strength straightly depends on the interphase and network strengths. A polymer nanocomposite contains the polymer host, nanoparticles, interphase, and filler network after the percolation onset.

Furthermore, the behavior of a nanocomposite obviously reflects the properties of its components. Consequently, the strength of a nanocomposite correlates to the strengths of its constituents, as expressed by the developed model. The direct dependence of nanocomposite strength on the strengths of interphase and filler net was designated at the prior studies [53,57]. Accordingly, the positive roles of interphase and net strengths in the nanocomposite strength are rational, which defend the right development of the Callister model assuming the interphase and percolating network.

**Figure 6:**
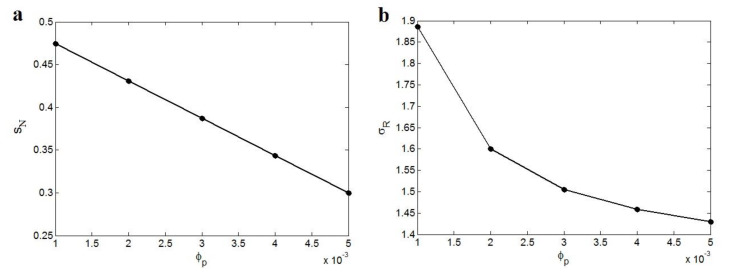
The effects of “$\varphi_{p}$ ” on (**a**) “*S_N_*” and (**b**) “*σ_R_*” at $\varphi_{f}$ = 0.02, *σ_m_* = 40 MPa, *R* = 10 nm, *t* = 20 nm, *N* = 300 and *σ_i_* = *σ_N_* = 5000 MPa.

Figure 6 shows the effects of “$\varphi_{p}$” on “*S_N_*” and “*σ_R_*” at $\varphi_{f}$ = 0.02, *σ_m_* = 40 MPa, *R* = 10 nm, *t* = 20 nm, *N* = 300 and *σ_i_* = *σ_N_* = 5000 MPa. The “*S_N_*” parameter decreases by an increment of “$\varphi_{p}$” at the same levels of other parameters. According to Figure 6a, *S_N_* = 0.47 is obtained at $\varphi_{p}$ = 0.001, while the “$\varphi_{p}$” level of 0.005 decreases the “*S_N_*” to 0.3, demonstrating the reverse relation of “$\varphi_{p}$” parameter with “*S_N_*”. “$\varphi_{p}$” determines the filler concentration above which the nanoparticles produce the networks. Clearly, a high “$\varphi_{p}$” delays the network creation to high filler concentrations. So, the observation of an inverse correlation between “$\varphi_{p}$” parameter and “*S_N_*” as a network parameter is not peculiar.

Figure 6b also exemplifies the “$\varphi_{p}$” significance on the relative strength of nanocomposites based on Equation (17). An opposite relation is also observed between “$\varphi_{p}$” parameter and relative strength of nanocomposites. The strongest sample is found by the least “$\varphi_{p}$”, where the high value of “$\varphi_{p}$” weakens the nanocomposites. As explained, a low “$\varphi_{p}$” indicates that the low concentration of nanoparticles can create a filler network. In this condition, the small amount of nanoparticles can mainly develop the nanocomposite strength through the formation filler network, which bears a high loading [58,59]. On the other hand, a high level of “$\varphi_{p}$” cannot generate a network in the nanocomposite by a low amount of nanoparticles. In this status, the nanocomposite cannot show a noteworthy tensile strength below percolation. Thus, the developed model decorously displays an inverse relation among the strength of nanocomposites and percolation onset.

## 4. Conclusions

A Callister model suggested for the strength of conventional composites was developed supposing the interphase and filler net roles by numerous parameters, including filler aspect ratio, percolation threshold, interphase thickness, and strength together with the density and strength of filler network. In this regard, two parameters including “*S_I_*” and “*S_N_*” were defined, which assume the interphase and network properties, correspondingly. The experimental results of several samples display a superior agreement with the developed model compared to the original one. The thinnest nanoparticles as well as the thickest interphase provide the finest levels of “*S_I_*” and tensile strength in polymer nanocomposites. Moreover, both “*N*” and “*α*” parameters directly manage the levels of “*S_N_*” and tensile strength of nanocomposite. Also, the high ranges of “*σ_m_*”, “*σ_i_*”, and “*σ_N_*” increase the “*S_I_*”, “*S_N_*”, and nanocomposite strength. The “*S_N_*” parameter decreases by an increment of “$\varphi_{p}$” at the same levels of other parameters, because a higher percolation threshold delays the development of net to greater filler concentration. An inverse relation is also observed between “$\varphi_{p}$” and relative strength of nanocomposites. In conclusion, the judicious correlations of tensile strength to the interphase and network possessions confirm the right expansion of Callister simulation for the tensile power of nanocomposite samples.
